# Relationship between sleep disturbances, lipid profile and insulin sensitivity in type 1 diabetic patients: a cross-sectional study

**DOI:** 10.20945/2359-3997000000228

**Published:** 2020-03-30

**Authors:** Ana Carolina Musser Tavares de Mattos, Yuri Sofiati Campos, Vitória Oliveira Fiorini, Yasmin Sab, Bruna Landeiro Tavares, Luis Guillermo Coca Velarde, Giovanna Aparecida Balarini Lima, Rubens Antunes da Cruz

**Affiliations:** 1 Departamento de Medicina Clínica Universidade Federal Fluminense Niterói RJ Brasil Departamento de Medicina Clínica, Universidade Federal Fluminense (UFF), Niterói, RJ, Brasil; 2 Departamento de Medicina Clínica Universidade Federal Fluminense Niterói RJ Brasil Curso de Pós-Graduação em Ciências Médicas, Departamento de Medicina Clínica, Universidade Federal Fluminense (UFF), Niterói, RJ, Brasil

**Keywords:** Sleep apnea, obstructive, diabetes mellitus, type 1, insulin resistance, dyslipidemias, extrinsic sleep disorder

## Abstract

**Objective:**

The consequences of sleep deprivation in type 1 diabetes (T1D) patients are poorly understood. Our aim was to determine how sleep disorders influence lipid profile and insulin sensitivity in T1D patients.

**Materials and methods:**

This was a cross-sectional study at a public university hospital. Demographic information and medical histories were obtained during regular scheduled visit of T1D patients to the outpatient clinic. Insulin sensitivity was obtained using the estimated glucose disposal rate (eGDR) formula. Sleep quality was assessed using the Pittsburgh Sleep Quality Index, Epworth Sleepiness Scale and Berlin Questionnaire.

**Results:**

The adult participants (n = 66, 62% women) had a median age of 28.0 years (interquartile range 21.8-33.0). Six patients (9%) had metabolic syndrome according to the International Diabetes Federation criteria. Thirty patients (46%) were considered poor sleepers according to the Pittsburgh Sleep Quality Index. The LDL-c and total cholesterol levels of poor sleepers were higher than those of good sleepers (103 v. 81; p = 0.003 and 178.0 v. 159.5 mg/dL; p = 0.009, respectively). Three patients (4%) were at high risk of obstructive sleep apnea syndrome (OSAS) according to the Berlin Questionnaire. The eGDR was lower in the group of patients with high probability of having OSAS (6.0 v. 9.1 mg.kg^-1^.min^-1^;p = .03).

**Conclusions:**

Poor subjective quality of sleep and higher risk of OSAS were correlated with a worsened lipid profile and decreased insulin sensitivity, respectively. Therefore, T1D patients with sleep disturbances might have an increased cardiovascular risk in the future.

## INTRODUCTION

Chronic disorders of sleep and wakefulness adversely affect health and daily functioning (1). However, considering the size of the problem, awareness among health care professionals and the general public is low (2). Patients with type 2 diabetes (T2D) present with sleep disturbances more frequently than individuals from the general population, and this is associated with an increase in the prevalence of T2D (3-5).

Considering type 1 diabetes (T1D), there are few studies describing sleep disorders and their consequences. Van Dijk and cols. reported that, compared with controls, adult patients with long standing T1D have disturbed subjective sleep quality and are at a higher risk for obstructive sleep apnea syndrome (OSAS) (6). Donga and cols. showed that partial sleep deprivation in adult T1D patients, even for a single night, reduced peripheral insulin sensitivity by 21% (7). In another study, the authors used a non-invasive validated score, based on waist circumference (WC), hypertension, and glycated hemoglobin A1c (HbA1c), that was developed to estimate the glucose disposal rate in T1D patients (8). This method allows a better estimation of insulin sensitivity compared with the gold standard test (the euglycemic hyperinsulinemic clamp) and is being considered a predictor of mortality in these individuals (9). A recent cross-sectional study investigated the relationship between sleep, circadian parameters, and insulin sensitivity as assessed by the estimated glucose disposal rate (eGDR) in 109 adults with T1D and demonstrated that poor sleep quality and sleep duration were negatively associated with eGDR independent of age, gender, smoking status, and body mass index (10).

These sleep disturbances in T1D patients may include lower sleep time percentage, long and short awake periods, and an increased number of awake periods (11). A study with young T1D patients reported that sleepiness and/or poor sleep habits correlated with reduced quality of life, depressed mood, lower grades at school, and lower reading scores. It is also considered that sleep should be routinely assessed as part of diabetes management in youth with T1D (12).

In the present study, we sought to determine whether adult patients with T1D have disturbances in subjective sleep characteristics assessed by validated sleep questionnaires. In addition, we also sought to determine if subjective sleep disturbances could be related to patient lipid profile and insulin sensitivity.

## MATERIALS AND METHODS

### Design and setting

This was a cross-sectional study performed with patients during their regular scheduled visits to a tertiary endocrinology outpatient clinic at a public university hospital. The individuals were recruited prospectively and randomly from June 2016 until August 2017. Subsequently, a medical interview was conducted, a detailed physical examination was performed, and blood samples were collected. All subjects entered the study after they had given their written informed consent in accordance with a protocol approved by the local Ethics Committee (protocol number: 1.623.289).

## Study design

### Data sources and patient population

Patients older than 18 years with a clinical diagnosis of T1D were included, and the T1D diagnosis was based on the following parameters: initiation of insulin therapy within the first year of diabetes diagnosis, past hospitalization for diabetic ketoacidosis, and/or a positive test for Anti-GAD (Glutamic Acid Descarboxilase antibodies). The exclusion criterion was the presence of any acute clinical condition that could temporarily interfere with the sleep pattern.

The investigator obtained the anthropometrical measurements – namely, the waist circumference (WC) and the hip circumference (HC). Waist-to-hip ratio (WHR) was calculated by dividing WC by HC. Body mass index (BMI) was calculated as weight in kilograms divided by the square of the height in meters (kg/m^2^) (13). Overweight and obesity were defined as BMI 25.0- 29.9 kg/m^2^ and BMI > 30 kg/m^2^, respectively.

Fasting glucose, total cholesterol, high-density lipoprotein cholesterol (HDL-c), and triglycerides (TG) were measured using commercial kits ^(^Labtest, Belo Horizonte, MG, Brazil) from blood samples obtained after a 12h overnight fast. Low-density lipoprotein cholesterol (LDL-c) was estimated using the Friedewald equation (14).

Insulin sensitivity was assessed using the eGDR (mg.kg^-1^.min^-1^) and was calculated as previously described according to the following formula:



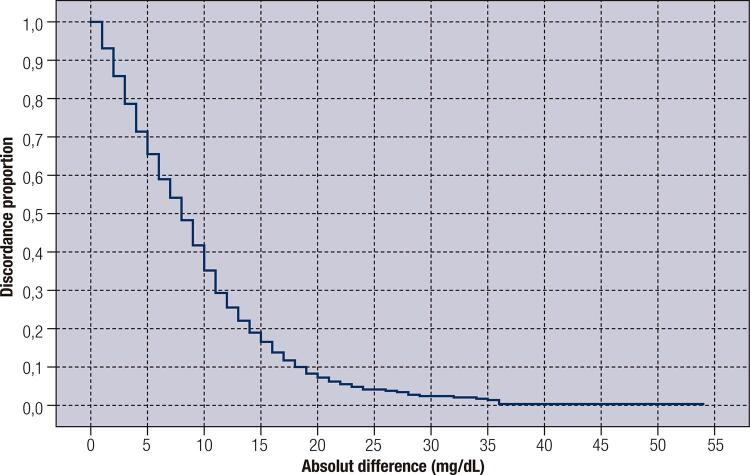



Where HT = hypertension (yes = 1/no = 0) and HbA1c = % HbA1c (8).

The reference range of HbA1c was 4-6% (Variant II, Bio-Rad) and was measured using high-pressure liquid chromatography. An appropriate glycemic control was considered when percentage HbA1c was ≤ 7.0%.

To evaluate the presence of metabolic syndrome (MS), we considered the International Diabetes Federation (IDF) consensus criteria, which require the presence of increased WC (≥ 80cm in women or ≥ 94 cm in men) and two of the following components: a) hypertension, defined as antihypertensive treatment and/or elevated blood pressure (systolic ≥ 130 mmHg or diastolic ≥ 85 mmHg); b) dyslipidemia, defined as elevated plasma TG (≥ 150 mg/dL) and/or low HDL-c cholesterol (< 40 mg/dL in men or < 50 mg/dL in women); c) fasting glucose ≥ 100 mg/dL or previous diagnosis of diabetes (14).

## Sleep questionnaires

### Pittsburgh Sleep Quality Index (PSQI)

The first method of indexing sleep quality was the Portuguese validated version of PSQI, the PSQI-BR (15-16). It uses a 19 item self-rated questionnaire for assessing subjective sleep quality over the month previous to the interview. The seven component scores of the PSQI are subjective sleep quality, sleep latency, sleep duration, habitual sleep efficiency, sleep disturbances, use of sleep medication, and daytime dysfunction. Each component is weighted from 0 to 3, and the global PSQI score ranges from 0 to 21. Participants with a score > 5 are considered poor sleepers, and higher scores indicate worsening sleep quality. Global PSQI score was estimated for each participant as described previously (15).

### Epworth Sleepiness Scale

This uses an eight-item questionnaire that focuses on the likelihood of falling asleep in several common situations encountered in daily life (17). Each question is scored on a progressive scale of intensity from 0 to 3, and the total score ranges from 0 (the least sleepy) to 24 (the most sleepy). Hypersomnolence or excessive daytime sleepiness (EDS) was considered when a score was equal to or above ten. In this study we used a validated Portuguese version of the Epworth Sleepiness Scale questionnaire (18).

### Berlin Questionnaire (BQ)

The BQ detects the risk of having sleep apnea. It consists of ten questions on risk factors for OSAS, subdivided into three symptom categories: 1) snoring and apneas (six points); 2) wake-time sleepiness or fatigue (three points); and 3) the presence of obesity or hypertension (two points). If two or more categories are positive a person is at high risk of having OSAS (categories 1 and 2: two or more points; category 3: one or more points). In this study we used a validated version of the BQ in Portuguese (19).

## Statistical analysis

The results are presented as medians and interquartile ranges (IQR) for continuous variables and counts with proportions for categorical variables. Associations between sleep characteristics and categorical variables were determined using the chi-square test. Associations between continuous variables and sleep characteristics were determined using the Mann-Whitney U test. The Spearman rank correlation coefficient (r_s_) was used to evaluate the correlation between numerical variables. Analyses were performed using SPSS version 23.0 for Windows (SPSS Inc., Chicago, IL, USA). p values < .05 were considered statistically significant.

## RESULTS

### Clinical characteristics

Sixty-six patients with T1D (41 women) were included. Only twenty-one (32%) patients engaged in regular physical activity, with a median of 4 days/week and 60 minutes (IQR 20-90) per day. Thirty (45%) patients were following a diet prescribed by a nutritionist. Seventeen (25%) patients were taking statins for dyslipidemia.

The median age was 28 years (IQR 21.7-33.0), and the median duration of T1D was 14 years (IQR 9.7-20.0). The median BMI was 23.3 kg/m^2^(IQR 21.6-25.4). Fourteen patients (21%) were overweight, and two patients (3%) were obese. Nine patients (14%) had MS according to the IDF criteria.

The median HbA1c was 8.3% (IQR 7.4%-10.1%), and nine (14%) patients had HbA1c ≤ 7%. A negative correlation between HbA1c level and daily insulin dosage (r_s _= -.40; p = .001) and years of formal education (r_s _= -.261; p= .02) was found. Moreover, there was a positive correlation between HbA1c level and LDL-c (r_s _= .27; p = .03), total cholesterol (r_s _= .37; p = .02), and TG levels (r_s _= .25; p = .04).

The median eGDR was 9.0 mg.kg^-1^.min^-1^ (IQR 7.4-10.2), and it presented a positive correlation with the number of years of formal education (r_s _= .35; p = .004) and a negative correlation with the duration of disease (r_s _= -.35; p = .005), total cholesterol level (r_s _= .30; p = .01), and TG level (r_s _= -.30; p = .01).

The clinical characteristics are summarized in Table 1.

**Table 1:** Clinical characteristics of the 66 T1D patients

Characteristic	Result
Age (years)	28.0 (21.7-33.0)
Formal education (years) Physical Activity (days)	11.0 (9.5-12.0) 4 (1 - 5)
BMI (kg/m^2^)	23.3 (21.6-25.4)
WC (cm)	78.0 (74.8-85.0)
eGDR (mg/kg/min)	9.0 (7.4-10.2)
HDL-c (mg/dL)	58.0 (49.0-69.0)
LDL-c (mg/dL)	91.5 (75.7-112.0)
Total cholesterol (mg/dL)	168.5 (150.0-195.8)
TG (mg/dL)	78.0 (51.7-101.0)
Insulin dosage (U/kg/day)	0.81 (0.56-1.03)

Data are presented as median (interquartile range).

BMI: body mass index; WC: waist circumference; eGDR: estimated glucose disposal ratio; TG: triglycerides; U: units.

### Sleep questionnaire analysis

#### PSQI-BR

The mean declared sleep duration among the 66 participants was 7.18 ± 1.89h, range = 4 to 12h. Thirty (46%) patients were classified as poor sleepers, and they had higher LDL-c and total cholesterol levels when compared with those of good sleepers (103 *vs. *81 mg/dL; p = .003 and 178.0 *vs.* 159.5 mg/dL; p = .009, respectively). Positive correlations were found between the PSQI-BR score and the LDL-c and total cholesterol levels (r_s _= .33; p = .007 and r_s _= .25; p = .048, respectively). No differences were found in terms of BMI, WC, disease duration, HbA1c levels, and eGDR.

#### Epworth Sleepiness Scale

Of all participants, 22 (33%) had EDS, with 16/22 (73%) being women. When comparing patients with and without EDS, no difference was found in any anthropometric or metabolic parameter.

#### BQ

Three patients (4%) were considered at high risk of having OSAS according to the BQ. All were women, and only one had MS. The eGDR was lower in the group of patients with high risk of having OSAS when compared with the rest of the patients (6.0 *vs.* 9.1 mg.kg^-1^.min^-1^; p = .03).

The correlation between the metabolic characteristics and the questionnaires scores are summarized in Table 2.

**Table 2:** Correlation between the questionnaires’ results and the metabolic characteristics of the T1D patients (n = 66)

Questionnaires	PSQI		ESE		BQ	

Result	> 5 (n = 30)	≤ 5 (n = 36)	≥ 10 (n = 22)	< 10 (n = 44)	High risk (n = 3)	Low risk (n = 63)
BMI (kg/m^2^)	22.8 (21.6-25.0)	23.1 (21.7-26.6)	22.8 (21.8-24.6)	23.1 (21.5-25.4)	23.4 (22.5-24.2)	22.9 (21.6-25.4)
eGDR (mg.kg^-1^.min^-1^)	9.0 (8.1-9.6)	9.1 (6.3-10.4)	9.1 (7.3-9.7)	9.0 (8.1-10.2)	6.0 (5.0-6.9)^c^	9.1 (8.0-10.2)
HDL-c (mg/dL)	58.0 (51.0-69.0)	57.5 (46.0-66.0)	58.0 (51.0-66.0)	57.5 (46.5-69.5)	53.0 (47.5-63.5)	58.0 (49.0-67.5)
LDL-c (mg/dL)	103.0 (90.0-117.0)^a^	81.0 (72.0-95.0)	92.0 (75.5-107.5)	91.0 (76.0-111.0)	89.0 (82.0-96.0)	91.5 (75.5-112.0)
TC (mg/dL)	178.0 (166.0-206.0)^b^	159.5 (139.5-181.5)	170.5 (150.0-185.0)	168.0 (150.5-202.0)	176.0 (153.5-181.0)	168.0 (150.5-196.5)
TG (mg/dL)	89.0 (63.0-101.0)	67.5 (44.0-99.0)	64.0 (40.5-95.0)	86.5 (58.0-101.0)	72.0 (67.5-110.5)	80.0 (51.5-101.0)

Data presented as median (interquartile range). BMI: body mass index; eGDR: estimated glucose disposal ratio; TC: total cholesterol; TG: triglycerides; U: units; PSQI-BR: Pittsburgh Sleep Quality Index; ESE: Epworth Sleepiness Scale; BQ: Berlin Questionnaire; EDS: Excessive Diurnal Sleepiness; OSAS: Obstructive Sleep Apnea Syndrome. ^a^ p < .003; ^b^ p < .009; ^c^ p < .03.

## DISCUSSION

Our results demonstrated that a high proportion of participants had sleeping problems with interference in the insulin sensitivity. We also observed that patients with bad sleep quality had increased LDL-c and total cholesterol levels.

We observed a high prevalence of patients without adequate glycemic control, with a median HbA1c of 8.3%. Previous studies had similar findings, which show that the difficulty in achieving an adequate HbA1c level is a worldwide issue (20-22). Along with this finding, only 14.8% of the patients had MS according to the IDF criteria. The use of intensive insulin therapy to optimize metabolic control has become generalized, and improved glycemic control results in increased weight gain and insulin resistance, thus increasing the frequency of overweight individuals and the risk of developing MS. The increase in the incidence of MS, along with a reduction in the occurrence of microvascular complications, has led cardiovascular disease to be considered the leading cause of death in T1D patients over 30 years of age (23).

The eGDR method has been widely used to evaluate, in a non-invasive way, the insulin sensitivity in patients with diabetes. Several studies recognize insulin resistance as an important feature in T1D, and it is considered a predictor of coronary artery disease (24,25). In our study, the eGDR was negatively correlated with two cholesterol components (TG and total cholesterol), indicating that reduced insulin sensitivity may be related to a worsened lipid profile. This is an important finding, as eGDR was considered an independent predictor of coronary artery disease in the Pittsburgh Epidemiology of Diabetes Complications study (26).

There are few studies in the literature evaluating the sleep quality in T1D patients, and ours is a pioneering study in Brazil. Regarding the PSQI, we found a high percentage of patients with T1D classified as poor sleepers, which corroborates the previous findings in literature (6). In a meta-analysis by Reutrakul and cols. analyzing the mean difference in sleep duration between T1D adults (n = 157) and controls (n = 9951), the T1D patients had a shorter sleep duration in the PSQI, with a mean difference of 0.73 minutes compared to controls, although the difference was not statistically significant (p = .3) (27). One benefit of this questionnaire is that it evaluates the subjective sleep impressions of the patients for the previous month, which diminishes the possibility of acute interferences in the sleep quality.

We observed a worse lipid profile in poor sleepers, with higher LDL-c and total cholesterol levels (p = .003; p = .009, respectively). We also found a statistically significant positive correlation between the PSQI score and higher total cholesterol levels (p = .007) in T1D patients. Our hypothesis is that, as already described in the literature, a poor sleep quality leads to a stress response, stimulating the hypothalamic-pituitary-adrenal axis, causing a hypersecretion of glucocorticoids (28). Jennings and cols. also found that lower PSQI scores are related to MS and several of its components, such as BMI and estimated insulin resistance (29). A recent study, conducted by Rosu and cols., found a similar result concerning poor sleep and decreased insulin sensitivity using eGDR levels but without the positive correlation between a PSQI and other MS components (10).

Regarding the Epworth Sleepiness Scale, more than one third of T1D patients had EDS, which is higher than its frequency in the general population (30). However, this finding had no correlation with any anthropometric or metabolic parameter.

Only three participants were considered at high risk of having OSAS. This finding might be explained by the characteristics of our sample, with a median BMI of 23.3 kg/m^2^. Although the number of patients with positive BQ scores was low, the eGDR of these patients was lower than that of the rest of the group, suggesting that T1D patients with higher risk for OSAS have a tendency to present decreased insulin sensitivity. Insulin resistance has been described as a consequence of OSAS, and it is being considered a manifestation of MS (31). Vgontzas and cols. found that OSAS is related to MS independently of obesity, and the reason is that sleep apnea may accelerate metabolic abnormalities, such as insulin resistance, possibly through elevation of stress hormones and cytokines such as cortisol, IL-6, and TNF-α (32,33). This reinforces our findings, as our patients with positive BQ scores were not obese.

This study has several limitations. First, data on depression was not collected even though it is a potentially significant reason for poor sleep quality, as we would not be have been able to define whether the results were associated with depression or emotional distress. Although it is commonly accepted that there is a high prevalence of depression among T1D patients, a review of the literature shows widespread inconsistencies in this diagnosis and its association with glycemic control (34,35). Second, the fact that 25% of our participants were using statins may have influenced the lipid profile results. Third, the cross-sectional design limited us to describe the association between sleep disturbances and metabolic parameters. Finally, although a small sample was included, there were significant associations between sleep disturbances, lipid profile, and insulin sensitivity. Additional longitudinal studies are needed to prove any causality in these associations.

In conclusion, poor sleep quality was associated with higher levels of LDL-c and total cholesterol in patients with T1D, and those at high risk for OSAS had decreased insulin sensitivity, even if they were not obese. Thus, T1D patients with sleep disturbances as determined by the questionnaires have a worse lipid profile and decreased insulin sensitivity, which may increase the risk of developing MS and consequently increase their cardiovascular risk in the future. The three questionnaires that were applied in this study are fast and easy to use and could add important information about the sleep quality of T1D patients. There are very few studies analyzing the effects of poor sleep quality and MS components in T1D; in Brazil, there has not been any such study. Therefore, further studies with a greater number of patients are necessary to corroborate these data.
